# Sedentary Time and Markers of Chronic Low-Grade Inflammation in a High Risk Population

**DOI:** 10.1371/journal.pone.0078350

**Published:** 2013-10-29

**Authors:** Joseph Henson, Thomas Yates, Charlotte L. Edwardson, Kamlesh Khunti, Duncan Talbot, Laura J. Gray, Thomas M. Leigh, Patrice Carter, Melanie J. Davies

**Affiliations:** 1 National Institute for Health Research (NIHR) Leicester-Loughborough Diet, Lifestyle and Physical Activity Biomedical Research Unit, Leicester, Leicestershire, United Kingdom; 2 Diabetes Research Centre, College of Medicine, Biological Sciences and Psychology, University of Leicester, Leicester, Leicestershire, United Kingdom; 3 Unilever Discover, Colworth Science Park, Sharnbrook, Bedfordshire, United Kingdom; 4 Department of Health Sciences, University of Leicester, Leicester, Leicestershire, United Kingdom; 5 University Hospitals of Leicester, Leicester Diabetes Centre, Leicester General Hospital, Leicester, Leicestershire, United Kingdom; University of South Florida College of Medicine, United States of America

## Abstract

**Background:**

Sedentary behaviour has been identified as a distinct risk factor for several health outcomes. Nevertheless, little research has been conducted into the underlying mechanisms driving these observations. This study aimed to investigate the association of objectively measured sedentary time and breaks in sedentary time with markers of chronic low-grade inflammation and adiposity in a population at a high risk of type 2 diabetes mellitus.

**Methods:**

This study reports data from an ongoing diabetes prevention programme conducted in Leicestershire, UK. High risk individuals were recruited from 10 primary care practices. Sedentary time (<25counts per 15s) was measured using Actigraph GT3X accelerometers (15s epochs). A break was considered as any interruption in sedentary time (≥25counts per 15s). Biochemical outcomes included interleukin-6 (IL-6), C-reactive protein (CRP), leptin, adiponectin and leptin:adiponectin ratio (LAR). A sensitivity analysis investigated whether results were affected by removing participants with a CRP level >10 mg/L, as this can be indicative of acute inflammation.

**Results:**

558 participants (age = 63.6±7.7years; male = 64.7%) had complete adipokine and accelerometer data. Following adjustment for various confounders, sedentary time was detrimentally associated with CRP (*β* = 0.176±0.057, *p* = 0.002), IL-6 (*β* = 0.242±0.056, *p* = <0.001), leptin (*β* = 0.146±0.043, *p* = <0.001) and LAR (*β* = 0.208±0.052, *p* = <0.001). Associations were attenuated after further adjustment for moderate-to-vigorous physical activity (MVPA) with only IL-6 (*β* = 0.231±0.073, *p* = 0.002) remaining significant; this result was unaffected after further adjustment for body mass index and glycosylated haemoglobin (HbA_1c_). Similarly, breaks in sedentary time were significantly inversely associated with IL-6 (*β* = −0.094±0.047, *p* = 0.045) and leptin (*β* = −0.075±0.037, *p* = 0.039); however, these associations were attenuated after adjustment for accelerometer derived variables. Excluding individuals with a CRP level >10 mg/L consistently attenuated the significant associations across all markers of inflammation.

**Conclusion:**

These novel findings from a high risk population recruited through primary care suggest that sedentary behaviour may influence markers associated with inflammation, independent of MVPA, glycaemia and adiposity.

## Introduction

Prospective and experimental studies in adults have postulated that chronic low-grade inflammation contributes to the pathogenesis of multiple chronic diseases, including type 2 diabetes mellitus (T2DM) [1]–[4].

Adipocytes are known to secrete large numbers of inflammatory cytokines, termed adipokines, which are directly affected by the levels and distribution of adiposity; consequently, obesity is considered a chronic inflammatory disease [5]–[8]. The levels and action of inflammatory adipokines are also thought to be mediated by moderate- to vigorous-intensity physical activity (MVPA) independent of adiposity [9]–[11].

Recent advances in physical activity research have suggested that sedentary behaviour, defined as any sitting activity with a low energy expenditure, is a distinct risk factor for several health outcomes [12]–[15]. Nevertheless, research examining the independent association between sedentary time and inflammatory markers remain limited. Recent cross-sectional epidemiological studies have suggested an association between sedentary behaviour and biomarkers linked to low-grade inflammation, independent of MVPA [16]–[18]. However, many of these associations were attenuated when adjusted for BMI and sedentary time has tended to be captured using self-report [17], [18], which has limited validity compared to objective measures, particularly for habitual non-structured activities [19].

Therefore, in order to further elucidate the role of sedentary behavior as a determinant of metabolic health, it is necessary to investigate the effect of objective measures of sedentary behaviour on inflammatory adipokines and to establish whether associations are independent of both MVPA and adiposity.

The purpose of this study was to examine those at a high risk of T2DM and the extent to which sedentary time and breaks in sedentary time are independently associated with IL-6, an adipokine directly involved in the inflammatory cascade and linked to the pathogenesis of many chronic diseases [20]. In addition, leptin (thought to be a modulator of the inflammatory response), adiponectin (a key anti-inflammatory adipokine), and C-reactive protein (CRP) are also investigated [21], [22].

## Methods

### Ethics Statement

Ethical approval was obtained from the Nottingham Research Ethics Committee. Written informed consent was provided by all participants and measurements were performed by trained staff according to standard operating procedures.

### Participants

The present analysis reports baseline data from the Walking Away from Type 2 Diabetes study, the methods of which have been published elsewhere [23]. A total of 833 participants at a high risk of T2DM were recruited through 10 primary care practices in Leicestershire, UK in 2010; analysis was subsequently conducted in 2013. Individuals at high risk of impaired glucose regulation (IGR) (composite of impaired glucose tolerance (IGT) and/or impaired fasting glycaemia (IFG)) or T2DM were identified using a modified version of the automated Leicester Risk Score, which was specifically designed to be administered in primary care [24]. An automated platform using existing medical records was used to rank individuals for diabetes risk using predefined weighted variables (age, gender, BMI, family history of T2DM and use of antihypertensive medication). Individuals scoring within the 90^th^ percentile in each practice were invited to take part in the study. This process has been shown to have reasonable sensitivity and specificity for identifying participants at a high risk of IGR [24]. All individuals were unaware of their diabetes risk status before entering the study.

Individuals were excluded from the study if they had established T2DM or were currently taking steroids.

### Sedentary Time Assessment

At the baseline visit, all eligible participants were asked to wear a tri-axial accelerometer, (Actigraph GT3X, Florida, USA), for a minimum of seven consecutive days during waking hours. These accelerometers translate raw accelerations into activity counts. Freedson cut-points were used to categorise an epoch as sedentary (<25 counts per 15 seconds) or MVPA (≥488 counts per 15 seconds) [25]. Breaks in sedentary time were defined as a transition from a sedentary (<25 counts per 15 seconds) to an active state (≥25 counts per 15 seconds) [12], [16], [26].

Non-wear time was defined as a minimum of 60 minutes of continuous zero counts and days with at least 600 minutes of wear time were considered valid [12], [26]. In order to be included in the analysis, participants required a minimum of any four valid days [27].

A commercially available data analysis tool (KineSoft version 3.3.75, Kinesoft, New Brunswick, Canada; www.kinesoft.org) was used to process the accelerometer data.

### Demographic, anthropometric and biochemical measurements

Information on medication, ethnicity and smoking status was obtained following an interview administered protocol conducted by a healthcare professional. Social deprivation was determined by assigning an Index of Multiple Deprivation (IMD) score to the participant’s resident area [28]. IMD scores are publically available continuous measures of compound social and material deprivation which are calculated using a variety of data including current income, employment status and previous education. Body weight, body fat % (Tanita TBE 611, Tanita, West Drayton, UK) and waist circumference (midpoint between the lower costal margin and iliac crest) were measured to the nearest 0.1 kg, 0.1% and 0.5 cm respectively.

Venous blood samples were obtained following an overnight fast. All assays were measured in the same laboratory using stable methodologies and conducted by individuals blinded to the patients' identity. Glycosylated haemoglobin (HbA_1c_) was analysed using the Bio-Rad Variant II HPLC system (Bio-Rad Clinical Diagnostics, Hemel Hempstead, UK). Plasma glucose was measured using a glucose oxidase method on the Beckman Auto Analyzer (Beckman, High Wycombe, UK). All participants underwent an oral glucose tolerance test. Diabetes, impaired fasting glycaemia (IFG) and/or impaired glucose tolerance (IGT) categories were determined based on WHO 2006 criteria [29]. Here, impaired glucose regulation (IGR) refers to a composite of IFG and/or IGT.

### Biomarker Measurements

CRP was analysed using a high sensitivity (Minimum Interpretation Limit  =  0.1 mg/L) HORIBA ABX clinical chemistry analyser. IL-6 was analysed using quantikine high-sensitivity enzyme-linked immonosorbent assays (R&D systems). Leptin was analysed using AlphaLISA no wash fluorescence immunoassay kits (Perkin Elmer). Adiponectin was quantified using a time-resolved fluorescence immunoassay (R&D systems antibodies) on the AutoDELFIA (Perkin Elmer Life Sciences). All ELISA and fluorescence immunoassays were conducted in replicate on the same sample and the average value obtained. If the intra- assay coefficient of variation exceeded 10% for leptin and 15% for adiponectin, the assay was repeated using the same technique. Similarly, the IL-6 assay was repeated if the concentration was >2 pg/ml and the coefficient of variation >20% or the concentration was <2 pg/ml and the coefficient of variation >25%.

An additional variable measuring the leptin:adiponectin ratio (LAR) was derived as it has been shown to be highly associated with insulin resistance [30].

### Statistical Analysis

All statistical analyses were conducted using IBM SPSS Statistics v20.0. Due to their skewed distribution, adiponectin, CRP, IL-6 and leptin were log-transformed.

Linear regression analysis was used to examine the independent associations of sedentary time and breaks in sedentary time with markers of chronic low-grade inflammation.

Data was adjusted for age, gender, smoking status, ethnicity, social deprivation, anti-hypertensive (Beta (*β*)-blockers, angiotensin-converting-enzyme (ACE) inhibitors), lipid lowering (statin, fibrates), aspirin, non-steroidal anti-inflammatory (NSAID) medication, family history of T2DM and accelerometer wear time (average minutes per day) (model 1). We additionally adjusted for MVPA time (average minutes per day) and the associations for breaks were examined having also adjusted for sedentary time (average minutes per day) (model 2). In order to examine the extent to which adiposity and glycaemia may attenuate any observed relationships, we further adjusted for BMI and HbA_1c_ (model 3).

Significant associations were followed up with interaction terms to assess differences in the strength of the associations between sedentary time and gender using a model adjusted for the above covariates. Participants were also categorised into two groups (active vs. inactive) according to their level of MVPA (dichotomised into low and high levels around the median) in order to assess whether associations were consistent across activity categories.

Sensitivity analyses were conducted to investigate whether results were affected if a different measure of adiposity (waist, % body fat) or glycaemia (2-h glucose) were used as covariates. In order to be consistent with previous studies [31], [32], a sensitivity analysis also investigated whether results were affected by removing participants with a CRP level >10 mg/L, as this may be indicative of acute inflammation [33].

All analyses were two-sided; p<0.05 was considered significant for main effects and p<0.1 was considered significant for interactions.

## Results

A total of 558 (67%) participants had complete adipokine and accelerometer data. The main reasons for participants not having complete data was insufficient accelerometer wear time over too few days and insufficient volumes of blood for additional analyses. There was no significant difference in the percentage of males/females, ethnic origin, BMI or social deprivation score in those included vs. excluded. However, those excluded were more likely to be younger (61.8±8.9 vs. 63.6±7.7years, p = 0.004). Table 1 reports the characteristics of the study participants.

**Table 1:** Participant characteristics.

**Variable**	
N	558
Age (years)	63.6±7.7
Male	364 (64.7)
Index-of-Multiple Deprivation score	13.0 (8.0–23.9)
Current smokers	45 (8.1)
Family History of Diabetes (1^st^ degree)	208 (37.3)
BMI (kg/m^2^)	32.2±5.2
Waist circumference (cm)	101.7±11.6
Weight (kg)	92.1±16.5
Body fat (%)	35.6±8.8
HbA_1c_ (%)	5.9 (5.6–6.1)
HbA_1c_ (mmol/mol)	41 (38–43)
2-hour plasma glucose (mmol/L)	6.1 (4.9–7.7)
High sensitivity C-reactive protein (mg/L)	1.6 (0.6–4.0)
Interleukin-6 (pg/mL)	2.0 (1.4–3.0)
Leptin (ng/mL)	9.0 (4.9–15.0)
Adiponectin (ug/ml)	11.3 (8.1–16.4)
Leptin/adiponectin ratio	0.8 (0.4–1.4)
**Blood pressure medication**	
β-blockers	96 (17.2)
Angiotensin-converting enzyme inhibitors	111 (19.9)
Aspirin medication	89 (15.9)
Non-steroidal anti-inflammatory medication	32 (5.7)
**Lipid lowering medication**	
Lipid lowering fibrates	2 (0.4)
Lipid lowering statins	187 (33.5)
**Diagnosis**	
Normal glucose tolerance	391 (70.1)
Isolated Impaired Fasting Glycaemia	26 (4.7)
Isolated Impaired Glucose Tolerance	96 (17.2)
Both	27 (4.8)
Diabetes	18 (3.2)
Impaired Glucose Regulation	149 (26.7)
Any abnormal glucose tolerance	167 (29.9)
**Ethnicity**	
White European	502 (90.0)
South Asian	40 (7.1)
Other	16 (2.9)
**Accelerometer variables (time in hours)**	
Time accelerometer worn	14.3±1.4
Sedentary Time	10.2±1.5
Moderate-to-vigorous physical activity	0.5 (0.3–0.8)
Breaks per day	269±64

Sedentary time  =  <25 counts per 15s and moderate-to-vigorous physical activity ≥488 counts per 15s. Continuous parametric results as mean±SD, number (column percentage) and continuous non-parametric results as median (interquartile range).

Table 2 displays the standardised regression coefficients for the association between sedentary time and breaks in sedentary time with markers of chronic low-grade inflammation.

**Table 2:** Associations of sedentary time and breaks in sedentary time with markers of chronic low-grade inflammation.

		**Model 1**		
	**Sedentary time**		**Breaks**	
	***β*** ** (SE)**	***p***	***β*** ** (SE)**	***p***
C-reactive protein	0.176 (0.057)	**0.002**	–0.088 (0.047)	0.064
Adiponectin	–0.098 (0.053)	0.068	0.062 (0.044)	0.159
Interleukin-6	0.242 (0.056)	**<0.001**	–0.094 (0.047)	**0.045**
Leptin	0.146 (0.043)	**0.001**	–0.075 (0.037)	**0.039**
Leptin/adiponectin ratio	0.208 (0.052)	**<0.001**	–0.079 (0.043)	0.064
		**Model 2**		
	**Sedentary time**		**Breaks**	
	***β*** ** (SE) ^a^**	***p***	***β*** ** (SE) ^b^**	***p***
C-reactive protein	0.093 (0.073)	0.205	–0.054 (0.065)	0.367
Adiponectin	–0.042 (0.069)	0.542	0.051 (0.060)	0.363
Interleukin-6	0.231 (0.073)	**0.002**	–0.024 (0.064)	0.694
Leptin	0.025 (0.056)	0.654	–0.053 (0.049)	0.243
Leptin/adiponectin ratio	0.030 (0.066)	0.650	–0.048 (0.052)	0.371
		**Model 3**		
	**Sedentary time**		**Breaks**	
	***β*** ** (SE) ^a^**	***p***	***β*** ** (SE) ^b^**	***p***
C-reactive protein	0.073 (0.072)	0.312	–0.032 (0.064)	0.587
Adiponectin	–0.040 (0.067)	0.551	0.044 (0.057)	0.421
Interleukin-6	0.212 (0.072)	**0.003**	–0.023 (0.063)	0.696
Leptin	0.026 (0.055)	0.634	–0.025 (0.044)	0.519
Leptin/adiponectin ratio	0.026 (0.064)	0.684	–0.027 (0.057)	0.580

**Model 1** was adjusted for age, gender, smoking status, ethnicity, social deprivation, family history, beta blockers, lipid lowering medication, aspirin, angiotensin-converting enzyme inhibitors, non-steroidal anti-inflammatory medication and time accelerometer worn.

**Model 2** was adjusted for the above covariates and ^a^moderate-to-vigorous physical activity or ^b^sedentary time and moderate-to vigorous physical activity.

**Model 3** was adjusted for the same covariates as Model 2 and BMI and HbA_1c._

### Sedentary time

Following adjustment for various confounders, sedentary time was detrimentally associated with IL-6 (*β* = 0.242±0.056, *p* = <0.001), CRP (*β* = 0.176±0.057, *p* = 0.002), leptin (*β* = 0.146±0.043, *p* = <0.001) and LAR (*β* = 0.208±0.052, *p* = <0.001). Associations were attenuated after adjustment for MVPA; with the exception of IL-6 (*β* = 0.231±0.073, *p* = 0.002), which remained significant after further controlling for BMI and HbA_1c_.

### Breaks in sedentary time

Independent of known confounders, breaks in sedentary time were significantly inversely associated with IL-6 (*β* =  –0.094±0.047, *p* = 0.045) and leptin (*β* =  –0.075±0.037, *p* = 0.039). However, these associations were attenuated when further adjusted for sedentary time and MVPA.

There were no significant interactions between gender and sedentary time or breaks in sedentary time across all markers of low-grade inflammation (all interactions p>0.1).

When the results were stratified by MVPA (active vs. inactive), sedentary time had a larger impact on IL-6 (*p* for interaction = 0.061) in the inactive group (Table 3). For breaks in sedentary time, the inactive cohort displayed a stronger association for CRP and HADP (*p* for interaction = <0.01).

**Table 3:** Associations of sedentary time and breaks in sedentary time with markers of chronic low-grade inflammation, stratified by MVPA (Active vs. Inactive).

	Active				Inactive				Interaction for sedentary time and MVPA	Interaction for breaks
	Sedentary time		Breaks^a^		Sedentary time		Breaks^a^			
	*β* (SE)	*p*	*β* (SE)	*p*	*β* (SE)	*p*	*β* (SE)	*p*	*p*	*p*
C-reactive protein	0.079 (0.085)	0.780	0.038 (0.086)	0.657	0.164 (0.108)	0.132	–0.120 (0.068)	0.199	0.352	**0.094**
Adiponectin	–0.036 (0.078)	0.644	–0.093 (0.078)	0.255	–0.128 (0.090)	0.146	0.138 (0.058)	0.082	0.234	**0.005**
Interleukin-6	0.081 (0.082)	0.327	–0.034 (0.068)	0.687	0.335 (0.106)	**0.002**	–0.074 (0.068)	0.314	**0.061**	0.779
Leptin	0.094 (0.066)	0.164	–0.010 (0.050)	0.857	0.007 (0.025)	0.931	–0.055 (0.053)	0.419	0.472	0.773
Leptin/adiponectin ratio	0.175 (0.073)	**0.018**	0.027 (0.054)	0.669	0.059 (0.099)	0.550	–0.080 (0.063)	0.204	0.143	0.160

Adjusted for age, gender, smoking status, ethnicity, social deprivation, family history, beta blockers, lipid lowering medication, aspirin, angiotensin-converting enzyme inhibitors, non-steroidal anti-inflammatory medication, time accelerometer worn and ^a^sedentary time.

### Sensitivity Analyses

Results reported above were unaffected if waist circumference or % body fat rather than BMI was used in Model 3. The pattern and significance of results were unaffected if 2-hour glucose (as opposed to HbA_1c_) was used in model 3; standardised beta-coefficients were consistently within 10% (data not shown). From the 558 subjects with CRP values, 47 (8.4%) had levels >10 mg/L. After exclusion of these participants, the standardised beta-coefficients were consistently weaker across all markers of chronic low-grade inflammation (Table S1).

## Discussion

In this sample of high risk individuals, sedentary time was positively associated with CRP and adipokines (IL-6, leptin and LAR) after controlling for various confounding variables. All of these associations were attenuated after further adjustment for MVPA, with the exception of IL-6, which remained significant when also adjusted for BMI and HbA_1c_. Associations with breaks in sedentary time and inflammatory markers were less consistent, with all results being attenuated after controlling for total sedentary time and MVPA. To our knowledge, this is the first study to examine the effect of objectively measured sedentary time and breaks in sedentary time on inflammatory adipokines linked to T2DM in a high risk population. Indeed, this study enhances previous studies conducted in the general population, whilst highlighting the potential importance of sedentary time as a distinct modifiable health risk behaviour.

The observation that IL-6 levels are positively associated with sedentary time after adjustment for MVPA, adiposity and glucose regulation is a novel finding and may suggest an independent link between sedentary time and low-grade inflammation. Furthermore, after stratifying by MVPA, the detrimental effects of sedentary time on IL-6 were stronger in those individuals who were classed as inactive; suggesting that the effects of sedentary time may be more relevant in those individuals who do not engage in sufficient levels of MVPA. These findings build upon previous research which demonstrated a link between self-reported sedentary behaviour and IL-6, independent of MVPA [18]. Furthermore, an increase in ambulatory activity (typically quantified as walking), is known to have a strong inverse correlation with sedentary behaviour [16], and has been shown to be associated with reduced IL-6 in those with IGT, independent of obesity [34].

IL-6 is an established inflammatory cytokine which is centrally involved in the inflammatory cascade and chronic elevations are a determinant of chronic disease and metabolic/vascular health. Low-grade inflammation, as determined by an elevated IL-6 level, has been shown to precede and be a distinct risk factor for the development of T2DM [35], [36]. As such, much research has focused upon the pleiotropic nature of IL-6 as it can induce anti-inflammatory effects in the acute-phase response [37], [38]. Specifically, IL-6 is a key myokine released from contracting muscle during exercise. The acute elevations in localised and systemic IL-6 during and immediately following exercise are thought to have anti-inflammatory effects and act to mediate the link between physical activity and metabolic health [1]. Prolonged sitting-related sedentary behaviour may therefore act to reduce the magnitude or otherwise impair this myokine response and thus contribute to a pro-inflammatory state [37]–[40]. Others have demonstrated in animal models that muscle immobilisation is linked to a blunted triglyceride uptake and low HDL-cholesterol levels [41], which in turn can influence particular cellular processes known to exacerbate chronic low-grade inflammation [42]. Nevertheless, experimental research specifically investigating changes to the myokine response following acute and chronic alterations in sedentary behaviour or light intensity ambulation, as opposed to MVPA, have yet to be conducted. Therefore, specific mechanisms linking sedentary behaviour to markers of inflammation remain unclear.

The association between sedentary time and IL-6 was attenuated after excluding participants with a CRP-level >10 mg/L. Therefore, caution needs to be applied when interpreting the results due to potential bias through acute inflammation. However, it has been argued that a CRP>10 mg/L lacks sensitivity when used to distinguish between acute and chronic inflammation [43]. Therefore, removal of such participants may exclude many with chronic inflammation who are at the highest risk for poor health outcomes [43]; particularly as high CRP values (>10 mg/L) are not uncommon and previous studies have demonstrated that >5% of the population exhibit such values at a given time-point [44], [45]. This may be particularly pertinent in individuals who exhibit characteristics such as obesity, smoking and physical inactivity, all known risk factors for chronic inflammation.

The non-significant results observed for breaks in sedentary time are in contrast to previous research carried out in the general population, which showed associations between breaks and CRP [16]. The discrepancy in results may be partly explained by the fact that our subjects spent a longer time in sedentary pursuits. Furthermore, the crude method used to quantify breaks in sedentary time may have precluded any associations, particularly as it was not possible to calculate the duration and intensity of each break. However, when the results were stratified by MVPA, breaks in sedentary time were more strongly associated with CRP and HADP in the inactive group. Therefore, breaking sedentary time may also be of greater relevance in individuals who do not reach the recommended levels of MVPA; however further research is needed in order to elucidate these findings.

This study has several strengths; it provides novel evidence in a high risk primary care population using an objective measure of sedentary time. Limitations include the cross-sectional design which complicates the drawing of causal inferences. It is also possible that unmeasured lifestyle or demographic variables were confounding the observed relationships. However, we adjusted for key medications and behaviours (smoking) known to affect inflammation and metabolic health. Furthermore, the relatively small sample size may have limited our statistical power and therefore precluded important significant observations compared to other studies [16].

## Conclusions

These novel findings from a high risk population based study recruited through primary care suggest that sedentary behaviour may influence some markers of adiposity-associated inflammation, independent of MVPA, glycaemia and anthropometric measures attributable to central adiposity. The deleterious effects of sedentary behaviour may be particularly pertinent for those individuals who do not undertake sufficient amounts of MVPA. However, further epidemiological and experimental evidence are needed to confirm these results and to elucidate specific mechanisms linking sedentary behaviour to chronic inflammation.

## Supporting Information

Table S1 Associations of sedentary time and breaks in sedentary time with markers of chronic low-grade inflammation (participants with CRP >10 mg/L removed).
(DOC)

## References

[1] Pedersen BK, Febbraio MA (2012) Muscles, exercise and obesity: Skeletal muscle as a secretory organ. Nat Rev Endocrinol 8: 457–465. 22473333 10.1038/nrendo.2012.49

[2] Wang X, Bao W, Liu J, Ouyang YY, Wang D, et al (2013) Inflammatory markers and risk of type 2 diabetes: A systematic review and meta-analysis. Diabetes Care 36: 166–175. 23264288 10.2337/dc12-0702 PMC3526249

[3] Kahn SE, Hull RL, Utzschneider KM (2006) Mechanisms linking obesity to insulin resistance and type 2 diabetes. Nature 444: 840–846. 17167471 10.1038/nature05482

[4] Tsigos C, Papanicolaou DA, Kyrou I, Defensor R, Mitsiadis CS, et al (1997) Dose-dependent effects of recombinant human interleukin-6 on glucose regulation. J Clin Endocrinol Metab 82: 4167–4170. 9398733 10.1210/jcem.82.12.4422

[5] Trayhurn P, Wood IS (2004) Adipokines: Inflammation and the pleiotropic role of white adipose tissue. Br J Nutr 92: 347–355. 15469638 10.1079/bjn20041213

[6] Franks PW, Farooqi IS, Luan J, Wong MY, Halsall I, et al (2003) Does physical activity energy expenditure explain the between-individual variation in plasma leptin concentrations after adjusting for differences in body composition? J Clin Endocrinol Metab 88: 3258–3263. 12843173 10.1210/jc.2002-021426

[7] Ertek S, Cicero A (2012) Impact of physical activity on inflammation: Effects on cardiovascular disease risk and other inflammatory conditions. Arch Med Sci 8: 794–804. 23185187 10.5114/aoms.2012.31614 PMC3506236

[8] Nader GA, Lundberg IE (2009) Exercise as an anti-inflammatory intervention to combat inflammatory diseases of muscle. Curr Opin Rheumatol 21: 599–603. 19726993 10.1097/BOR.0b013e3283319d53

[9] Gleeson M, Bishop NC, Stensel DJ, Lindley MR, Mastana SS, et al (2011) The anti-inflammatory effects of exercise: Mechanisms and implications for the prevention and treatment of disease. Nat Rev Immunol 11: 607–615. 21818123 10.1038/nri3041

[10] Kasapis C, Thompson PD (2005) The effects of physical activity on serum C-reactive protein and inflammatory markers: A systematic review. J Am Coll Cardiol 45: 1563–1569. 15893167 10.1016/j.jacc.2004.12.077

[11] Panagiotakos DB, Pitsavos C, Chrysohoou C, Kavouras S, Stefanadis C, et al (2005) The associations between leisure-time physical activity and inflammatory and coagulation markers related to cardiovascular disease: The ATTICA study. Prev Med 40: 432–437. 15530595 10.1016/j.ypmed.2004.07.010

[12] Henson J, Yates T, Biddle SJ, Edwardson CL, Khunti K, et al (2013) Associations of objectively measured sedentary behaviour and physical activity with markers of cardiometabolic health. Diabetologia 56: 1012–1020. 23456209 10.1007/s00125-013-2845-9

[13] Edwardson CL, Gorely T, Davies MJ, Gray LJ, Khunti K, et al (2012) Association of sedentary behaviour with metabolic syndrome: A meta-analysis. PLoS One 7: e34916. 22514690 10.1371/journal.pone.0034916 PMC3325927

[14] Thorp AA, Owen N, Neuhaus M, Dunstan DW (2011) Sedentary behaviors and subsequent health outcomes in adults a systematic review of longitudinal studies, 1996–2011. Am J Prev Med 41: 207–215. 21767729 10.1016/j.amepre.2011.05.004

[15] Wilmot EG, Edwardson CL, Achana FA, Davies MJ, Gorely T, et al (2012) Sedentary time in adults and the association with diabetes, cardiovascular disease and death: Systematic review and meta-analysis. Diabetologia 55: 2895–2905. 22890825 10.1007/s00125-012-2677-z

[16] Healy GN, Matthews CE, Dunstan DW, Winkler EA, Owen N (2011) Sedentary time and cardio-metabolic biomarkers in US adults: NHANES 2003-06. Eur Heart J 32: 590–597. 21224291 10.1093/eurheartj/ehq451 PMC3634159

[17] Allison MA, Jensky NE, Marshall SJ, Bertoni AG, Cushman M (2012) Sedentary behavior and adiposity-associated inflammation: The multi-ethnic study of atherosclerosis. Am J Prev Med 42: 8–13. 22176840 10.1016/j.amepre.2011.09.023 PMC3244676

[18] Yates T, Khunti K, Wilmot EG, Brady E, Webb D, et al (2012) Self-reported sitting time and markers of inflammation, insulin resistance, and adiposity. Am J Prev Med 42: 1–7. 22176839 10.1016/j.amepre.2011.09.022

[19] Neilson HK, Robson PJ, Friedenreich CM, Csizmadi I (2008) Estimating activity energy expenditure: How valid are physical activity questionnaires? Am J Clin Nutr 87: 279–291. 18258615 10.1093/ajcn/87.2.279

[20] Pickup JC, Crook MA (1998) Is type II diabetes mellitus a disease of the innate immune system? Diabetologia 41: 1241–1248. 9794114 10.1007/s001250051058

[21] Tataranni PA, Ortega E (2005) A burning question: Does an adipokine-induced activation of the immune system mediate the effect of overnutrition on type 2 diabetes? Diabetes 54: 917–927. 15793228 10.2337/diabetes.54.4.917

[22] Lago R, Gomez R, Lago F, Gomez-Reino J, Gualillo O (2008) Leptin beyond body weight regulation—current concepts concerning its role in immune function and inflammation. Cell Immunol 252: 139–145. 18289518 10.1016/j.cellimm.2007.09.004

[23] Yates T, Davies MJ, Henson J, Troughton J, Edwardson C, et al (2012) Walking away from type 2 diabetes: Trial protocol of a cluster randomized controlled trial evaluating a structured education programme in those at high risk of developing type 2 diabetes. BMC Fam Pract 13: 46. 22642610 10.1186/1471-2296-13-46 PMC3444401

[24] Gray LJ, Davies MJ, Hiles S, Taub NA, Webb DR, et al (2012) Detection of impaired glucose regulation and/or type 2 diabetes mellitus, using primary care electronic data, in a multiethnic UK community setting. Diabetologia 55: 959–966. 22231125 10.1007/s00125-011-2432-x

[25] Freedson PS, Melanson E, Sirard J (1998) Calibration of the computer science and applications, inc. accelerometer. Med Sci Sports Exerc 30: 777–781. 9588623 10.1097/00005768-199805000-00021

[26] Healy GN, Dunstan DW, Salmon J, Cerin E, Shaw JE, et al (2008) Breaks in sedentary time: Beneficial associations with metabolic risk. Diabetes Care 31: 661–666. 18252901 10.2337/dc07-2046

[27] Trost SG, McIver KL, Pate RR (2005) Conducting accelerometer-based activity assessments in field-based research. Med Sci Sports Exerc 37: S531–43. 16294116 10.1249/01.mss.0000185657.86065.98

[28] The English Indices of Deprivation 2007 Summary. Available: http://webarchive.nationalarchives.gov.uk/20120919132719/http://www.communities.gov.uk/documents/communities/pdf/576659.pdf. Accessed 2013 July 19.

[29] World Health Organization 2006: Definition and diagnosis of diabetes mellitus and intermediate hyperglycaemia. Available: http://www.who.int/diabetes/publications/diagnosis_diabetes2006/en/index.html. Accessed 2013 July 19.

[30] Finucane FM, Luan J, Wareham NJ, Sharp SJ, O'Rahilly S, et al (2009) Correlation of the leptin:Adiponectin ratio with measures of insulin resistance in non-diabetic individuals. Diabetologia 52: 2345–2349. 19756488 10.1007/s00125-009-1508-3 PMC2759015

[31] Lee CC, Adler AI, Sandhu MS, Sharp SJ, Forouhi NG, et al (2009) Association of C-reactive protein with type 2 diabetes: Prospective analysis and meta-analysis. Diabetologia 52: 1040–1047. 19326095 10.1007/s00125-009-1338-3

[32] Best LG, Zhang Y, Lee ET, Yeh JL, Cowan L, et al (2005) C-reactive protein as a predictor of cardiovascular risk in a population with a high prevalence of diabetes: The strong heart study. Circulation 112: 1289–1295. 16116058 10.1161/CIRCULATIONAHA.104.489260

[33] Ridker PM (2003) Clinical application of C-reactive protein for cardiovascular disease detection and prevention. Circulation 107: 363–369. 12551853 10.1161/01.cir.0000053730.47739.3c

[34] Yates T, Davies MJ, Gorely T, Talbot D, Bull F, et al (2010) The effect of increased ambulatory activity on markers of chronic low-grade inflammation: Evidence from the PREPARE programme randomized controlled trial. Diabet Med 27: 1256–1263. 20950383 10.1111/j.1464-5491.2010.03091.x

[35] Hu FB, Meigs JB, Li TY, Rifai N, Manson JE (2004) Inflammatory markers and risk of developing type 2 diabetes in women. Diabetes 53: 693–700. 14988254 10.2337/diabetes.53.3.693

[36] Kristiansen OP, Mandrup-Poulsen T (2005) Interleukin-6 and diabetes: The good, the bad, or the indifferent? Diabetes 54 Suppl 2S114–24. 16306329 10.2337/diabetes.54.suppl_2.s114

[37] Heinrich PC, Castell JV, Andus T (1990) Interleukin-6 and the acute phase response. Biochem J 265: 621–636. 1689567 10.1042/bj2650621 PMC1133681

[38] Fischer CP, Hiscock NJ, Penkowa M, Basu S, Vessby B, et al (2004) Supplementation with vitamins C and E inhibits the release of interleukin-6 from contracting human skeletal muscle. J Physiol 558: 633–645. 15169848 10.1113/jphysiol.2004.066779 PMC1664960

[39] Pedersen M, Steensberg A, Keller C, Osada T, Zacho M, et al (2004) Does the aging skeletal muscle maintain its endocrine function? Exerc Immunol Rev 10: 42–55. 15633585

[40] Pedersen BK (2012) Muscular interleukin-6 and its role as an energy sensor. Med Sci Sports Exerc 44: 392–396. 21799452 10.1249/MSS.0b013e31822f94ac

[41] Hamilton MT, Hamilton DG, Zderic TW (2007) Role of low energy expenditure and sitting in obesity, metabolic syndrome, type 2 diabetes, and cardiovascular disease. Diabetes 56: 2655–2667. 17827399 10.2337/db07-0882

[42] Piya MK, McTernan PG, Kumar S (2013) Adipokine inflammation and insulin resistance: The role of glucose, lipids and endotoxin. J Endocrinol 216: T1–T15. 23160966 10.1530/JOE-12-0498

[43] Ishii S, Karlamangla AS, Bote M, Irwin MR, Jacobs DR Jr, et al (2012) Gender, obesity and repeated elevation of C-reactive protein: Data from the CARDIA cohort. PLoS One 7: e36062. 22558327 10.1371/journal.pone.0036062 PMC3340402

[44] Visser M, Bouter LM, McQuillan GM, Wener MH, Harris TB (1999) Elevated C-reactive protein levels in overweight and obese adults. JAMA 282: 2131–2135. 10591334 10.1001/jama.282.22.2131

[45] Alley DE, Seeman TE, Ki Kim J, Karlamangla A, Hu P, et al (2006) Socioeconomic status and C-reactive protein levels in the US population: NHANES IV. Brain Behav Immun 20: 498–504. 16330181 10.1016/j.bbi.2005.10.003

