# Supplementary material for: Sedentary Time and Markers of Chronic Low-Grade Inflammation in a High Risk Population
Source: PLoS One. 2013 Oct 29;8(10):e78350. doi: 10.1371/journal.pone.0078350 (PMC3812126; doi:10.1371/journal.pone.0078350)
Supplement: Table S1 — Associations of sedentary time and breaks in sedentary time with markers of chronic low-grade inflammation (participants with CRP >10 mg/L removed). (DOC) [file pone.0078350.s001.doc]

Table S1. Associations of sedentary time and breaks in sedentary time with markers of chronic low-grade inflammation (participants with CRP >10mg/L removed)

|  |  | **Model 1** |  |  |
| --- | --- | --- | --- | --- |
|  | **Sedentary time** |  | **Breaks** |  |
|  | ***β* (SE)** | ***p*** | ***β* (SE)** | ***p*** |
| C-reactive protein | 0.140 (0.058) | **0.017** | -0.053 (0.049) | 0.287 |
| Adiponectin | -0.076 (0.055) | 0.168 | 0.053 (0.046) | 0.333 |
| Interleukin-6 | 0.135 (0.058) | **0.022** | -0.074 (0.050) | 0.142 |
| Leptin | 0.122 (0.046) | **0.008** | -0.065 (0.038) | 0.202 |
| Leptin/adiponectin ratio | 0.189 (0.054) | **0.001** | -0.058 (0.047) | 0.217 |
|  |  | **Model 2** |  |  |
|  | **Sedentary time** |  | **Breaks** |  |
|  | ***β* (SE) ^a^** | ***p*** | ***β* (SE) ^b^** | ***p*** |
| C-reactive protein | 0.062 (0.076) | 0.417 | -0.019 (0.066) | 0.776 |
| Adiponectin | -0.012 (0.075) | 0.873 | 0.045 (0.062) | 0.395 |
| Interleukin-6 | 0.121 (0.077) | 0.127 | -0.026 (0.066) | 0.692 |
| Leptin | 0.057 (0.059) | 0.270 | -0.046 (0.051) | 0.237 |
| Leptin/adiponectin ratio | 0.008 (0.066) | 0.905 | -0.040 (0.061) | 0.515 |
|  |  | **Model 3** |  |  |
|  | **Sedentary time** |  | **Breaks** |  |
|  | ***β* (SE) ^a^** | ***p*** | ***β* (SE) ^b^** | ***p*** |
| C-reactive protein | 0.054 (0.076) | 0.481 | -0.001 (0.033) | 0.997 |
| Adiponectin | -0.002 (0.086) | 0.981 | 0.026 (0.061) | 0.670 |
| Interleukin-6 | 0.119 (0.077) | 0.119 | -0.010 (0.065) | 0.887 |
| Leptin | 0.051 (0.050) | 0.386 | -0.024 (0.043) | 0.578 |
| Leptin/adiponectin ratio | 0.003 (0.073) | 0.967 | -0.010 (0.056) | 0.860 |

**Model 1** was adjusted for age, gender, smoking status, ethnicity, social deprivation, family history, beta blockers, lipid lowering medication, aspirin, angiotensin-converting enzyme inhibitors, non-steroidal anti-inflammatory medication and time accelerometer worn

**Model 2** was adjusted for the above covariates and ^a^moderate-to-vigorous physical activity or ^b^sedentary time and moderate-to vigorous physical activity

**Model 3** was adjusted for the same covariates as Model 2 and BMI and HbA_1c_
